# Trust and 2024 Public Priorities for the CDC and State Health Departments

**DOI:** 10.1001/jamahealthforum.2024.0862

**Published:** 2024-05-24

**Authors:** Gillian K. SteelFisher, Mary G. Findling, Hannah L. Caporello, Alyssa Boyea, Laura Espino, Jazmyne Sutton

**Affiliations:** 1 Department of Health Policy and Management, Harvard T. H. Chan School of Public Health, Boston, Massachusetts; 2 Association of State and Territorial Health Officials, Arlington, Virginia; 3 National Public Health Information Coalition, Canton, Georgia; 4 SSRS, Glen Mills, Pennsylvania

## Abstract

This survey study evaluates public health priorities and trust in the Centers for Disease Control and Prevention (CDC) and state health departments among US adults after the COVID-19 pandemic.

## Introduction

Amid post–COVID-19 pandemic budget and workforce constraints, US public health agencies are challenged with addressing a diverse array of issues among an increasingly polarized public.^1,2,3,4,5^ Understanding how issues are seen by the public overall and among segments more and less likely to heed agency recommendations may help agencies tailor their approaches more effectively.^3^ This survey study examined public priorities for agencies, comparing perspectives of those with higher and lower levels of trust.

## Methods

We conducted a survey from November 3 to 20, 2023, among a nationally representative, probability-based sample of US adults aged 18 years or older via internet and telephone. This study was determined exempt by the Harvard T. H. Chan School of Public Health institutional review board per the Common Rule. Participants provided consent through the survey. We followed the AAPOR reporting guideline (details in the eMethods in Supplement 1).

Respondents reported levels of trust in health recommendations of the Centers for Disease Control and Prevention (CDC) and their state health department (SHD) and indicated whether they thought each of 11 issues (drawn from SHD agendas across the country^4^) should be a top priority or lower priority or should not be addressed by the CDC or SHD. We compared respondents’ priorities across segments with different levels of trust in the CDC and SHDs using bivariate analyses to provide a frame for public health leaders given evidence of the centrality of trust as a factor associated with people’s response to the COVID-19 pandemic in this dataset and in the broader literature.^3,6^ Differences in priorities were examined using 2-tailed *t* tests in Stata, version 15.0. Data were weighted to US census parameters to produce nationally representative estimates (eTable in Supplement 1).

## Results

Among 5594 invited participants, 2663 (48%) completed the survey. Most expressed at least some trust in public health agencies’ health recommendations (CDC: 75%; SHD: 79%) (Figure). Less than one-quarter had little or no trust in agencies’ recommendations (CDC: 24%; SHD: 21%).

**Figure.**
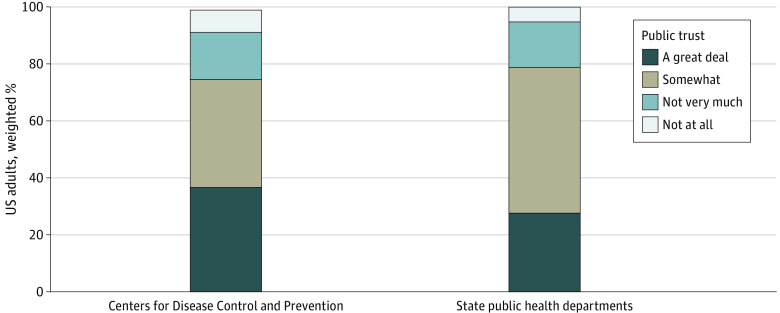
Public Trust in Centers for Disease Control and Prevention and State Health Department Health Recommendations After the COVID-19 Pandemic, November 2023 Data are from a November 2023 nationally representative, probability-based online and telephone survey of 2663 US adults aged 18 years or older. The eMethods in Supplement 1 gives details. The full question was, “In terms of recommendations made to improve health in general, how much do you trust the recommendations of each of the following groups …The Centers for Disease Control and Prevention, or CDC? … Your state public health department?” Percentages may not add to 100% due to rounding.

Nine of 11 issues were deemed a top priority by more than half, including 4 by about three-quarters or more: preventing chronic diseases (CDC: 83%; SHD: 79%), preventing and addressing mental illness (CDC: 80%; SHD: 85%), reducing infant mortality (CDC: 79%; SHD: 78%), and preventing and addressing opioid and other substance addiction (CDC: 72%; SHD: 77%) (Table). These 4 were deemed top priorities among a majority in every trust level. Four issues were particularly divisive, with large gaps (36-64 percentage points) in the proportion deeming them top priorities when comparing those with the highest and lowest trust levels: controlling COVID-19, controlling non–COVID-19 infectious disease spread, addressing racial and ethnic disparities, and preventing gun injuries.

**Table.** Top US Public Priorities for the Centers for Disease Control and Prevention (CDC) and State Public Health Departments to Address Overall and by Degree of Trust, November 2023^a^

Top priorities	US adults, by level of trust, weighted %									
	CDC					State health department				
	All (n = 1325)	A great deal (n = 521)	Somewhat (n = 485)	Not very much (n = 219)	Not at all (n = 100)	All (n = 1338)	A great deal (n = 371)	Somewhat (n = 675)	Not very much (n = 221)	Not at all (n = 71)
Preventing chronic diseases like heart disease, cancer, and diabetes	83^b^	85^c^	84^c^	82^c^	71	79^b^	81	80	75	72
Preventing and addressing mental illness	80^b^	84^c^	79^c^	77^c^	64	85^b^	87^d^	87^d^	79	78
Reducing infant mortality	79^b^	82^c^	76^c^	84^c^	59	78^b^	78	78	79	70
Preventing and addressing opioid and other substance addiction	72^b^	74	72	71	70	77^b^	81^d^	77^d^	67	80
Controlling the spread of infectious diseases other than COVID-19	72^e^	86^c^^,^^d^^,^^f^	70^c^^,^^d^	58^c^	41	65^e^	76^c^^,^^d^^,^^f^	64^c^	57^c^	40
Reducing differences in health status and health care access between people in different racial or ethnic groups	59^e^	73^c^^,^^d^^,^^f^	57^c^^,^^d^	46^c^	24	65^e^	81^c^^,^^d^^,^^f^	65^c^^,^^d^	42	41
Reducing death and illness related to HIV and other sexually transmitted infections	58^e^	70^c^^,^^d^^,^^f^	52	49	43	52^e^	63^c^^,^^d^^,^^f^	51^d^	40	44
Preventing obesity and promoting healthy diets and physical activity	58^e^	61^c^	55	62^c^	46	57^b^	59	56	57	51
Controlling the spread of COVID-19	57^e^	79^c^^,^^d^^,^^f^	55^c^^,^^d^	30^c^	15	51^e^	70^c^^,^^d^^,^^f^	50^c^^,^^d^	31^c^	17
Preventing injuries and deaths caused by guns	49^g^	65^c^^,^^d^^,^^f^	49^c^^,^^d^	27	17	59^e^	78^c^^,^^d^^,^^f^	60^c^^,^^d^	34	28
Preventing negative health outcomes from cigarettes and e-cigarettes	47^g^	52^d^^,^^f^	44	42	40	47^g^	59^c^^,^^d^^,^^f^	45^d^	34	37

^a^ Data are from a November 2023 nationally representative, probability-based, online and telephone survey of 2663 US adults aged 18 years or older. Statistical significance was considered at *P* < .05. Statistically significant results should be interpreted with caution, as some analyses were conducted with fewer than 100 respondents. The eMethods in Supplement 1 give details.

^b^ A majority of adults cited this issue as a top agency priority across all levels of trust.

^c^ Higher than those who trust public health agencies “not at all.”

^d^ Higher than those who trust public health agencies “not very much.”

^e^ A majority of adults overall cited this issue as a top agency priority but not across all levels of trust.

^f^ Higher than those who trust public health agencies “somewhat.”

^g^ A majority of adults overall did not cite this issue as a top agency priority.

## Discussion

This study found that after the COVID-19 pandemic, there was wide public support for action on parts of health agency agendas^4^ even among a polarized public. Multiple topics on the public health agenda had majority support, including several with majority support from the least trusting: chronic diseases, mental health, infant mortality, and opioids and addiction. While public health agencies must navigate public views of specific activities, knowing which issues are more universally embraced may provide opportunities to showcase some activities and, longer term, may build public support for institutions as people see their priority issues addressed. Knowing there is wide public support may help agencies garner support from bipartisan stakeholders for their efforts.

The results showed less universal support for prioritizing COVID-19 or other infectious diseases after the pandemic. Public health agencies should plan for additional communication when addressing these and other important issues, including racial and ethnic disparities and gun injuries.

Study limitations include use of cross-sectional, self-reported data and the potential for nonresponse bias. Public opinion data demonstrated that federal and state public health agencies’ opportunities for action and gaining trust have not been entirely diminished after the COVID-19 pandemic, though thoughtful approaches will still be required in areas of division.

## Notes

Supplement 1. eMethods.

Supplement 2. Data Sharing Statement
