# Supplementary material for: Trust and 2024 Public Priorities for the CDC and State Health Departments
Source: JAMA Health Forum. 2024 May 24;5(5):e240862. doi: 10.1001/jamahealthforum.2024.0862 (PMC11127117; doi:10.1001/jamahealthforum.2024.0862)
Supplement: Supplement 1. — eMethods. [file jamahealthforum-e240862-s001.pdf]

Supplemental Online Content

SteelFisher GK, Findling MG, Caporello HL, Boyea A, Espino L, Sutton J. Trust and 2024 public priorities for the CDC and state health departments. *JAMA Health Forum.* Published online May 24, 2024. doi:10.1001/jamahealthforum.2024.0862

eMethods.

This supplemental material has been provided by the authors to give readers additional information about their work.

© 2024 SteelFisher GK et al. *JAMA Health Forum.*

A. eMethods: Questionnaire Development and Question Language

Details on Questionnaire Development

The questionnaire was developed using American Association of Public Opinion Research best practices for survey research.[1] The content and wording of questions, response options, and question order and flow were all developed after reviewing prior surveys, and the list of 11 public health issues was generated from an environmental scan of public health agencies' agendas.[2]

Respondents were randomized to be asked about priorities for either their state health department (half sample A) or Centers for Disease Control and Prevention (CDC) (half sample B). Before fielding, the questionnaire was reviewed for bias, balance, and comprehension, with pretesting among a small of participants to improve the survey quality.

Questions	Response Categories
(Asked of all, n=2,663) In terms of recommendations made to improve health in general, how much do you trust the recommendations of each of the following groups?<br>a. The Centers for Disease Control and Prevention, or CDC<br>b. Your state public health department<br>c. Your local public health department, meaning your city, county, or regional health department	A great deal<br>Somewhat<br>Not very much<br>Not at all
Ask of a randomized half-sample of respondents (Half Sample A, n=1338): In your view, should each of the following issues be a top priority for your state public health department to address in the next year, a lower priority for them to address, or should your state public health department not address this issue at all?<br><br>Ask of a randomized half-sample of respondents (Half Sample B, n=1325): In your view, should each of the following issues be a top priority for the Centers for Disease Control and Prevention, or CDC to address in the next year, a lower priority for the CDC to address, or should the CDC not address this issue at all?<br><br>1. Controlling the spread of COVID-19<br>2. Controlling the spread of infectious diseases other than COVID-19<br>3. Preventing and addressing mental illness<br>4. Preventing and addressing opioid and other substance addiction<br>5. Reducing infant mortality, or deaths among babies<br>6. Preventing chronic diseases like heart disease, cancer, and diabetes<br>7. Preventing obesity and promoting healthy diets and physical activity<br>8. Reducing death and illness related to HIV and other sexually transmitted infections<br>9. Reducing differences in health status, and health care access between people in different racial or ethnic groups<br>10. Preventing injuries and deaths caused by guns<br>11. Preventing negative health outcomes from cigarettes and e-cigarettes	Top priority<br>Lower priority<br>Should not address at all

[1] American Association for Public Opinion Research (AAPOR). Best Practices for Survey Research [Internet]; 2022. https://www.aapor.org/Standards-Ethics/Best-Practices.aspx#best3

[2] Association of State and Territorial Health Officials (ASTHO). Association of State and Territorial Health Officials' Annual Environmental Scan, 2022. https://www.astho.org/globalassets/pdf/environmental-scan.pdf

© 2024 SteelFisher GK et al. *JAMA Health Forum.*

B. eMethods: Sampling, Weighting, and Margin of Sampling Error Details

Respondents for the poll were reached through the SSRS Opinion Panel,[3] a nationally representative probability-based web panel. Given that this is a probability-based panel, findings are statistically projectable to the general U.S. adult population. A total of 2,502 surveys were completed by panelists online, and 161 interviews were completed by phone with panelists who do not have access to the internet, or who do not use it. The survey was administered in two stages: first among a sample of 1,031 respondents via the SSRS Opinion Panel Omnibus platform November 3-6, 2023, and then among a sample of 1,632 adults from November 10-20, 2023.

The sample was weighted in stages. The first stage of the weighting was the application of a base weight to account for different selection probabilities and response rates across sample strata. In the second stage sample demographics were post-stratified to match population parameters. Comparisons between the survey sample and national benchmarks for key demographics are shown in the next section. The design effect for this survey was 1.5, which means the margin of error for the entire sample is ±2.3 percentage points at the 95% confidence interval.

The participation rate for this survey was expected due to the rapid response nature of fielding the survey, as it was conducted as part of technical assistance for state and local health departments. Prior research suggests that the resulting data are comparable to longer-term, higher-response surveys when reweighted to key population parameters.[4] After weighting, differences between the survey sample and national sources for all demographic characteristics were $\leq$ 1 percentage point (see eTable 1 below).

[3] https://ssrs.com/opinion-panel/

[4] See, for example, Kohut et al. Assessing the Representativeness of Public Opinion Surveys. Pew Research Center. 2012. http://www.people-press.org/2012/05/15/assessing-the-representativeness-of-public-opinion-surveys; Mercer A, Lau A. Comparing Two Types of Online Survey Samples. Pew Research Center; 2023. https://www.pewresearch.org/methods/2023/09/07/comparing-two-types-of-online-survey-samples/; Keeter S, Hatley N, Kennedy C, Lau A. What Low Response Rates Mean for Telephone Surveys. Pew Research Center; 2017. https://www.pewresearch.org/wp-content/uploads/2017/05/RDD-Non-response-Full-Report.pdf.

© 2024 SteelFisher GK et al. *JAMA Health Forum.*

C. eTable 1: Comparison Between Sample and National Benchmarks for Key Demographics

eTable 1 below shows key demographics of the sample compared to benchmark data.

- Gender, age, education, race and ethnicity, and region benchmarks were derived from 2023 Current Population Survey (CPS)[5]. In the survey, gender age, education, and race and ethnicity were self-reported by participants.
- The civic engagement benchmark was derived from September 2021 Volunteering and Civic Life Supplement data[6] from the Current Population Survey (CPS).
- The population density came from Claritas Pop-Facts Premier 2023[7].
- The internet frequency benchmark is from the National Public Opinion Reference Survey (NPORS) for Pew Research Center - May 19 to Sept 5, 2023[8].

Weights were trimmed to prevent individual interviews from having too much influence on final results.

As shown in eTable 1 below, differences between the weighted sample and benchmarks were all ≤ 1 percentage point.

eTable 1. Comparison Between Sample and National Benchmarks for Key Demographics

Variable		Benchmark %	Weighted %
Gender	Male	49.0%	48.7%
	Female	51.0%	51.3%
Age (in years)	18-24	11.8%	11.4%
	25-34	17.4%	17.4%
	35-44	16.9%	17.0%
	45-64	31.5%	31.7%
	65+	22.4%	22.5%
Education	Less than HS	9.4%	8.9%
	HS diploma	28.8%	29.0%
	Some College	26.4%	26.5%
	College+	35.4%	35.6%
Region	North East	17.4%	17.4%
	Midwest	20.5%	20.6%
	South	38.6%	38.5%
	West	23.6%	23.4%
Civic Engagement	Engaged	27.0%	27.4%
	Not engaged	73.0%	72.6%
Race and Ethnicity	African American/Black, Non-Hispanic/Latinx	12.1%	12.1%
	Asian, Non-Hispanic/Latinx	6.5%	6.4%
	Hispanic/Latinx, Native Born	8.4%	8.6%
	Hispanic/ Latinx, Foreign Born	9.1%	8.5%
	White, Non-Hispanic/Latinx	61.3%	61.9%
	Other, Non-Hispanic/Latinx [9]	2.6%	2.5%
Density Quintiles	1	20.0%	20.0%
	2	20.0%	20.2%
	3	20.0%	20.0%
	4	20.0%	20.1%
	5	20.0%	19.8%
Internet Use Frequency	Almost constantly	42.0%	42.7%
	Several times a day	44.1%	44.3%
	Less often/Not an Internet User	13.9%	12.9%

[5] Sarah Flood, Miriam King, Renae Rodgers, Steven Ruggles, J. Robert Warren, Daniel Backman, Annie Chen, Grace Cooper, Stephanie Richards, Megan Schouweiler and Michael Westberry. IPUMS CPS: Version 11.0 [dataset]. Minneapolis, MN: IPUMS, 2023. https://doi.org/10.18128/D030.V11.0

[6] Civically engaged respondents are defined as those who have volunteered in the past 12 months or who talk to their neighbors daily. See the US Census Bureau: https://www.census.gov/programs-surveys/cps/about/supplemental-surveys.html

[7] See Claritas Pop-Facts Premier: https://environicsanalytics.com/data/demographic/pop-facts-premier

[8] See Pew Research Centers, https://www.pewresearch.org/methods/fact-sheet/national-public-opinion-reference-survey-npors/

[9] "Other, Non-Hispanic/Latinx" includes respondents who reported their race as multiracial; American Indian or Alaska Native; Native Hawaiian or Other Pacific Islander; or indicated they are another race that is not: African American or Black; Asian; American Indian or Alaska Native; Native Hawaiian or Other Pacific Islander; or White.

© 2024 SteelFisher GK et al. *JAMA Health Forum.*
