# Supplementary material for: Trust and 2024 Public Priorities for the CDC and State Health Departments
Source: JAMA Health Forum. 2024 May 24;5(5):e240862. doi: 10.1001/jamahealthforum.2024.0862 (PMC11127117; doi:10.1001/jamahealthforum.2024.0862)
Supplement: Supplement 2. — Data Sharing Statement [file jamahealthforum-e240862-s002.pdf]

Data Sharing Statement

SteelFisher. Trust and 2024 Public Priorities for the CDC and State Health Departments. *JAMA Health Forum*. Published May 24, 2024. doi:10.1001/jamahealthforum.2024.0862

Data
Data available: No

Additional Information
Explanation for why data not available: The authors are not making these data available because additional publications from these data are forthcoming.
